# HRRD: a manually-curated database about the regulatory relationship between HPV and host RNA

**DOI:** 10.1038/s41598-020-76719-6

**Published:** 2020-11-11

**Authors:** Bingqing Yan, Siwei Zhang, Siyang Yu, Sajjad Hussain, Tianyang Liu, Bozhi Wang, Xiaoyu Dong, Fen Ma, Lanlan Wei

**Affiliations:** 1 Department of Microbiology, Harbin Medical University, Harbin, 150081 China; 2 Wu Lien-Ten Institute, Harbin, 150081 China

**Keywords:** Bioinformatics, Human papilloma virus

## Abstract

HPV (Human papilloma virus) is a kind of small double-stranded DNA viruses which is extremely associated with different cancers. The roles HPV plays in the host were gradually identified through the interaction between it (including its early genes) and host RNA. In recent years, increasing numbers of studies in HPV-related cancers have been published showing the relationship between HPV and host RNA. Here, we present a database named HRRD, which contains the regulatory relationship between HPV and RNA (mRNA, miRNA and lncRNA). The information was extracted from 10,761 papers in PubMed (up to December 1st, 2019). In addition, the sequence map of HPV (198 genotypes) is also contained. HRRD was designed as a user-friendly web-based interface for data retrieval. It integrated the information of interaction between HPV and RNA, which reflects the relationship between HPV and host. We hope HRRD will further provide a comprehensive understanding of HPV in carcinogenesis and prognosis. HRRD is freely accessible at www.hmuhrrd.com/HRRD.

## Introduction

Human papilloma viruses (HPVs) are a group of double-stranded circular DNA viruses with about 8000 nucleotides in size. More than 200 HPV types have been characterized to date, including some high-risk types (16, 18, 31, 33 and so on). HPV genome is composed of six early gene (E1, E2, E4, E5, E6 and E7) open reading frames (ORFs), two late gene (L1 and L2) ORFs and a non-coding long control region (LCR). Distinct genes perform different functions^1^. After HPVs infect the basal cells of epithelia, they employ cellular machinery to replicate and survive in host cells^2^. With the persistent infection, HPV and the released viral genome will lead to squamous intraepithelial lesions, even cancers^3^, such as cervical cancer^4^ and head and neck cancer^5^. HPV not only plays an important role in carcinogenesis, but also participate in host tumor immunity, prognosis, drug resistance and so on. All these functions are achieved through the interaction between HPV and host RNA.

**Figure 1:**
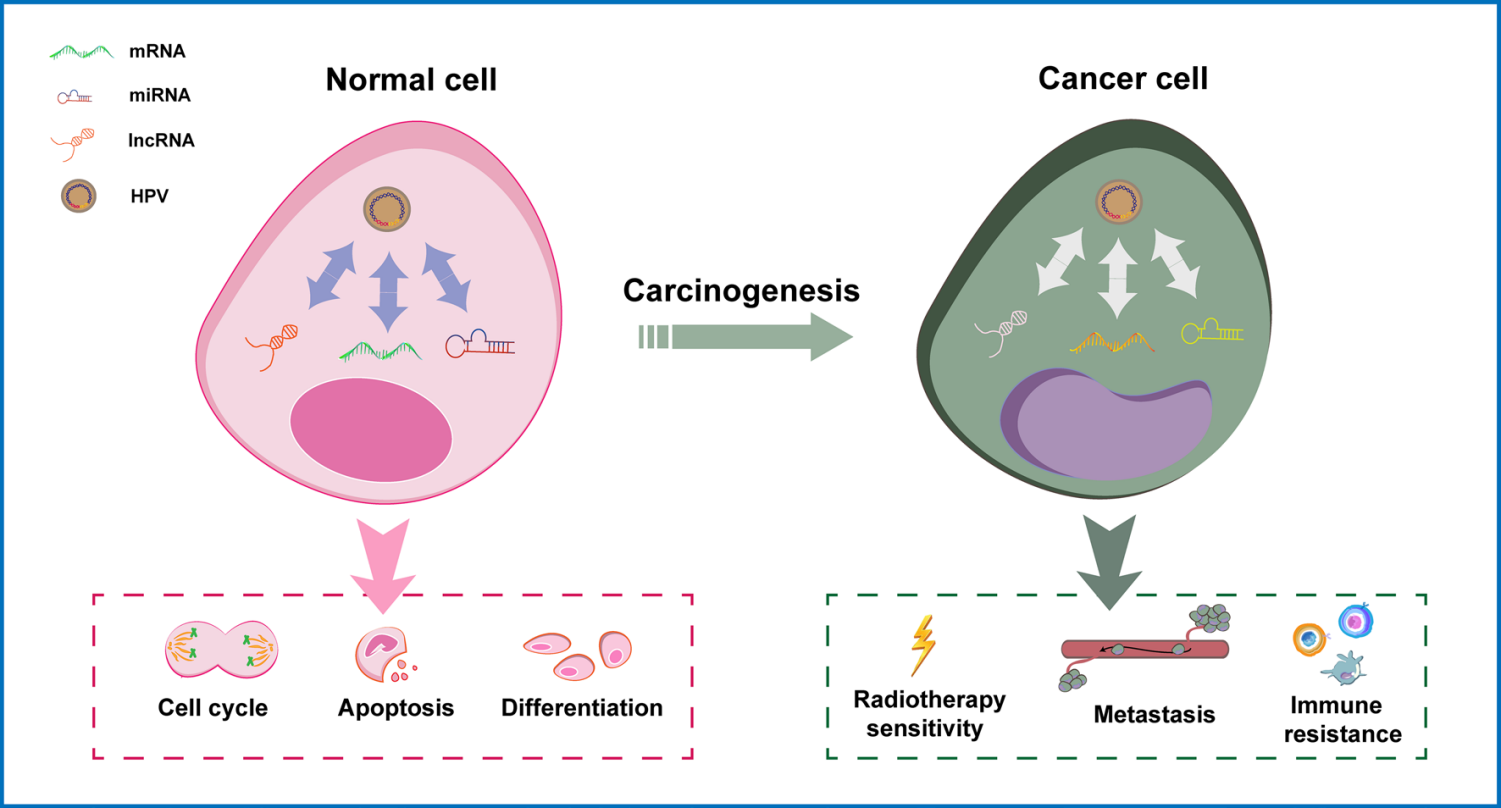
Patterns of Interaction between HPV and Host Cells via RNA. In normal cells, HPV influences cell cycle and differentiation and causes cancers via RNA. In cancer cells, HPV affects metastasis and prognosis via RNA.

Host RNA can be divided into coding RNA and non-coding RNA. mRNA is the main type of coding RNA, while miRNA and lncRNA are two kinds of non-coding RNA which are popular in the field of research. HPVs influence host cells (normal/cancer cells) by regulating the expression of these RNAs (Fig. 1). In normal cells, HPV mainly affects cell cycle, apoptosis and differentiation. For example, E6 promotes p53 degradation and inhibits cell apoptosis, while E7 binds to pRb and promotes cell growth and differentiation^6^. HPV activates miR-34 to participate in the cell apoptosis^7^. In addition, the expression of HOTAIR was significantly down-regulated by E7 to cause cervical cancer^8^. In cancer cells, HPV participates in cancer metastasis, prognosis, immune response and so on. For instance, HPV activates TGF-β and IL-10 expression to activate the host immunity^9^. HPV promotes tumor metastasis by affecting various miRNAs in HPV-related cancers^10^. Besides, there are many other RNA changes caused by HPV and its oncogenes in HPV-related cancers. By affecting the expression of host RNA, HPV can lead to the occurrence and development of cancers, and has a huge impact on the treatment, immunity and prognosis of cancers. Moreover, it has also been reported recently that host RNA can affect the expression of HPV in converse.

In the recent past, some databases about HPV have been published. PaVE^11^ is a database about papilloma virus (including HPV). HPVdb^12^ is a Human Papillomavirus T cell Antigen Database. HPVbase^13^ shows the integration information of HPV. However, these databases only focus on the biological characteristics of HPV itself, such as its type and sequence information, but do not collect any information about the HPV-infected host. Despite of numerous experimental studies in the field, there are no computational resources with a unique focus on the relationship between HPV and host RNA. Hence, it is important and necessary to establish a proper multi-comparative platform to show this mutual relationship. Here, we present HRRD, a database of the relationship between HPV and host RNA, which contains the existing studies on HPV and host RNA. It will contribute to providing a comprehensive web-based resource for HPV and host, and data support for HPV-related research.

## Methods

### Implementation

**Figure 2:**
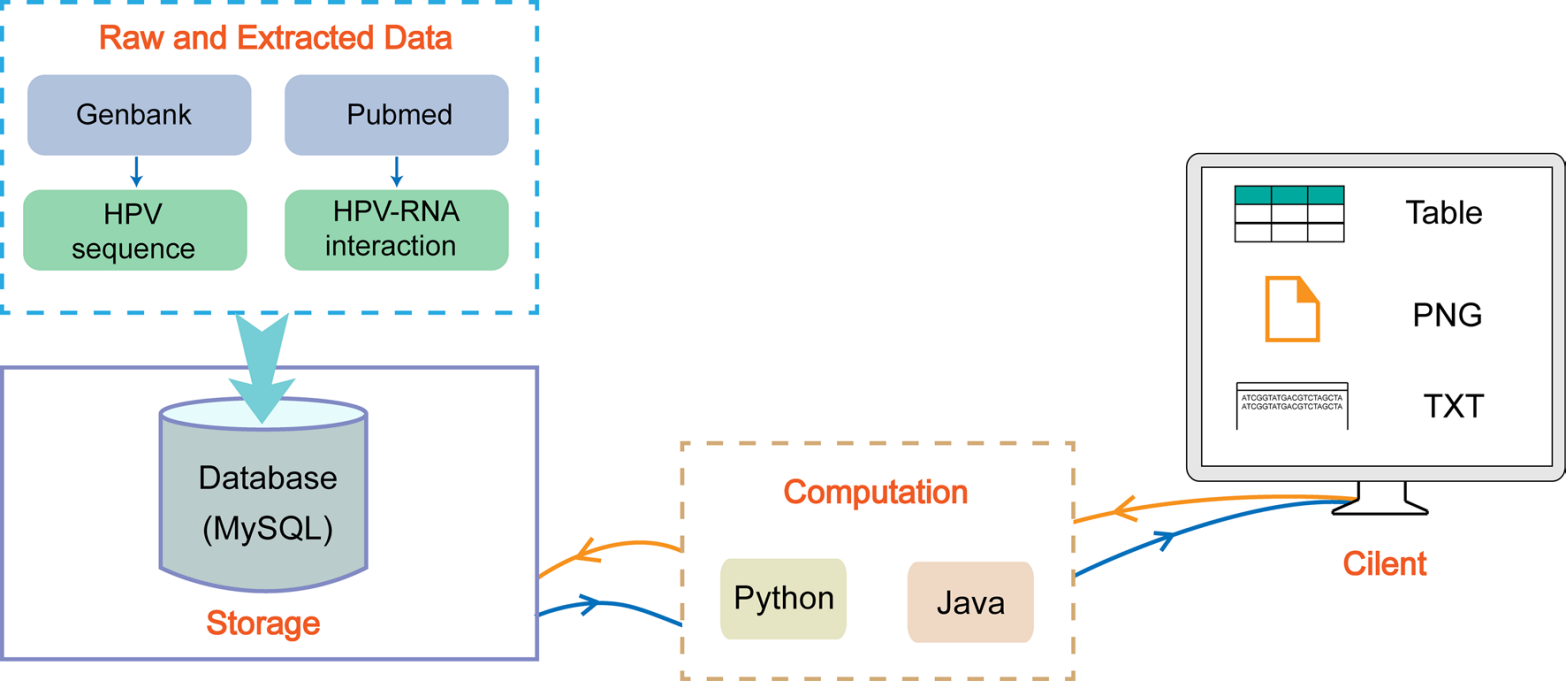
Flow chart of database construction. Files of HPV sequence were downloaded from Genbank. Related literatures searched by keywords were downloaded via web crawler in Pubmed. HPV-RNA interaction information was extracted manually. All the information was stored in HRRD, then computed or analyzed using scripts with outputs visualized in figures, txt and tables.

HRRD was developed with Eclipse (https://www.eclipse.org), one of the open-source integrated development environment (IDE). The database manage system was MySQL (https://www.mysql.com) which stores the relationship data model. Java Server Pages (JSP) in the web tier was used to present dynamic responses to clients. The client tier was made up of static and dynamic html pages including forms, tables, charts, and embedded JavaScripts. The model-view-controller (MVC) model web application was managed by Struts (https://struts.apache.org). All plots and web crawler were generated with python (version 3.8.1). HRRD is implemented and hosted on a Windows operating system, with Tomcat and MySQL (version 5.6.19), for serving static and dynamic content and providing the features for data management, respectively (Fig. 2).

### Data collection and processing

HPV-related search keywords include: HPV, human papillomavirus and human papilloma virus. RNA-related search keywords include: RNA, miRNA (microRNA), mRNA, lncRNA (long non-coding RNA), gene. Articles with the combination of keywords were searched on PubMed (excluding reviews and epidemiological studies). Web crawler got the research literatures with the keywords above. After deleting the epidemiological survey, review and the duplications, there were 10,761 articles remaining (up to December 1st, 2019). The whole genome information of HPV (198 genotypes with specific sequence information) was downloaded from NCBI in genbank format.

The information of the relationship between HPV and host RNA was extracted from articles, containing the PMID number, cancer type, HPV genotype, HPV oncogene, RNA type and name, method, material and the regulatory relationship between HPV and host RNA (action direction & action type). The information is obtained by reading the literature manually. The collected data were checked and unified, and finally summarized into Excel form. There are totally 784 pieces of information extracted from the articles, including 13 pieces of lncRNA, 195 pieces of miRNA, and others are mRNA.

The sequence information of different HPV was extracted from the genbank files, including the full-length genome of HPV and the location of its oncogenes. The information of HPV sequence was stored in the database in TXT format.

## Results

### Relationship page

**Figure 3:**
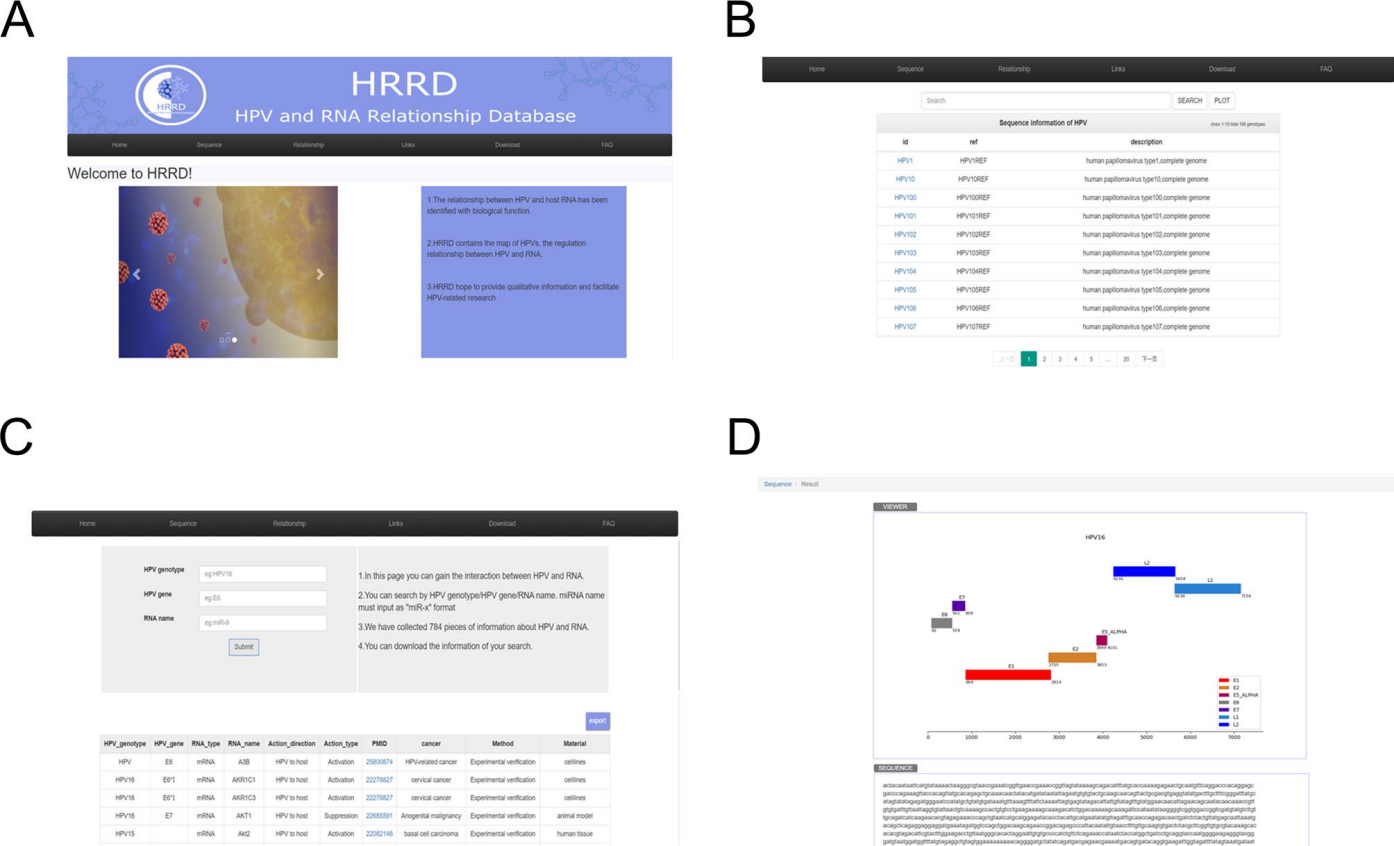
Display of the pages in the database. (**A**) Home page of HRRD. (**B**) Sequence page of HPV16 in HPV page. (**C**) Relationship page of HRRD. (**D**) The Sequence-result page of HRRD.

The principal function of HRRD is query. The information about the relationship between HPV and host RNA is stored in the database. In the Relationship page, there are three input boxes with examples for users to search. Users can search by one to three conditions like HPV genotype, HPV gene or RNA name to get a list containing the relevant content of users searched. The list would contain following information: HPV genotype, HPV gene, RNA type and name, action direction and type, cancer type, PMID, method and material. Users can jump to the article summary in PubMed by clicking the PMID number. “HPV to host” means HPV affects host RNA expression, while “host to HPV” means host RNA influences HPV expression. “Activation” refers to up-regulated expression, while “Suppression” means down-regulated expression. The method shows whether this message was concluded from experimental verification or data analysis (Fig. 3C). The result was sorted by the name of RNA. The export button can help users to export the searched results in XLS format.

### Sequence page

The genome information of HPVs is stored in the database. In the Sequence page, the table shows all species of HPV stored in HRRD. Users can input different genotype of HPV in the search box and click the search button to search HPV in the table below. Users can go to the result page by the plot button or clicking the HPV ID in the table (Fig. 3B). The result page is divided into two parts. The upper part displays the linear viewer of the corresponding HPV sequence, and the complete sequence of the HPV is shown in the lower part (Fig. 3D). The picture and sequence of HPV genome can be accessed directly.

### Other pages

Other pages contain some other ancillary functions. A simple Home page was designed (Fig. 3A) with a brief introduction of HRRD. The scrolling picture on the Home page shows HPV, the process of HPV infection and the effect of HPV on host cells. Some frequently asked questions and answers about HRRD are given in the FAQ page. In Links page, there shows the hyperlinks and brief introduction of some HPV-related databases like PaVE, HPVdb, HPVbase and NCBI. If necessary, users can visit the corresponding website. In the Download page, there is a software hyperlink named DisV-HPV16 (version I), which was developed for detecting HPV16 and its oncogenes expression in RNA-sequencing data.

## Discussion and future development

To our knowledge, HRRD is the first database designed for the relationship between HPV and RNA specifically. To ensure the accuracy of data, the information was manually extracted from relevant literature. HRRD describes the sequence map of HPV and incorporates some links to HPV-related databases so that users can search and get relevant information quickly and easily. We chose RNA as our research object, because RNA is unstable and susceptible to changes and mutates by viruses, and it is the first step in protein synthesis. For example, if researchers detect HPV infection in cancer, HRRD can help to find out whether HPV affects the key RNA and causes the carcinogenesis. In addition, if researchers want to find some key RNA in HPV-related cancer, HRRD can help to do preliminary screening and query. With the plenty resources, it will provide candidate biomarkers for HPV-related carcinogenesis and prognosis and identify potential targets for molecular therapy. We anticipate it will facilitate an insight into HPV and host.

Despite the database has contained data up to December 1st, 2019, a rapidly growing number of relevant researches are emerging from then. HRRD will be updated regularly by scanning newly published literatures. To ensure the timeliness of data, we plan to use data mining and machine learning to obtain information from the literatures. At the same time, we will update the software with modified function and expanded the scope of application. These updates will be implemented in the near future.

## Data Availability

HRRD is freely accessible at www.hmuhrrd.com/HRRD.
